# Perceptions of nurses working with psychiatric consumers regarding the elimination of seclusion and restraint in psychiatric inpatient settings and emergency departments: An Australian survey

**DOI:** 10.1111/inm.12522

**Published:** 2018-07-18

**Authors:** Adam Gerace, Eimear Muir‐Cochrane

**Affiliations:** ^1^ College of Nursing and Health Sciences Flinders University Adelaide South Australia Australia; ^2^ School of Health, Medical and Applied Sciences Central Queensland University Adelaide South Australia Australia

**Keywords:** acute inpatient units, emergency departments, mechanical restraint, physical restraint, psychiatric consumers, seclusion

## Abstract

Seclusion and restraint continue to be used across psychiatric inpatient and emergency settings, despite calls for elimination and demonstrated efficacy of reduction initiatives. This study investigated nurses’ perceptions regarding reducing and eliminating the use of these containment methods with psychiatric consumers. Nurses (*n = *512) across Australia completed an online survey examining their views on the possibility of elimination of seclusion, physical restraint, and mechanical restraint as well as perceptions of these practices and factors influencing their use. Nurses reported working in units where physical restraint, seclusion, and, to a lesser extent, mechanical restraint were used. These were viewed as necessary last resort methods to maintain staff and consumer safety, and nurses tended to disagree that containment methods could be eliminated from practice. Seclusion was considered significantly more favourably than mechanical restraint with the elimination of mechanical restraint seen as more of a possibility than seclusion or physical restraint. Respondents accepted that use of these methods was deleterious to relationships with consumers. They also felt that containment use was a function of a lack of resources. Factors perceived to reduce the likelihood of seclusion/restraint included empathy and rapport between staff and consumers and utilizing trauma‐informed care principles. Nurses were faced with threatening situations and felt only moderately safe at work, but believed they were able to use their clinical skills to maintain safety. The study suggests that initiatives at multiple levels are needed to help nurses to maintain safety and move towards realizing directives to reduce and, where possible, eliminate restraint use.

## Introduction

Seclusion and restraint—restricting a consumer's movement using environmental, physical, or mechanical means—are containment methods used with psychiatric consumers in inpatient settings and emergency departments (EDs) to prevent and manage the risk of harm because of behaviours such as aggression, violence, and self‐injury. These containment practices have been identified as involving deleterious physical and psychological effects for consumers and staff, and complex legal and ethical issues are associated with their use (McSherry 2017; Muir‐Cochrane & Gerace, 2014). In Australia and internationally, there have been continued calls to reduce and move towards elimination of these coercive practices (Department of Health, 2008; National Mental Health Consumer & Carer Forum, 2009; Substance Abuse and Mental Health Services Administration, 2017; The Royal Australian and New Zealand College of Psychiatrists, 2016).

Reduction in seclusion and restraint use has been documented in Australia (Australian Institute of Health and Welfare, 2018). However, seclusion, physical, and mechanical restraint remain relatively common practices, with recent studies highlighting concerning factors such as the use of these practices multiple times with the same consumers (Oster *et al*. 2016) or for prolonged periods of time (McKenna *et al*., 2017). This highlights an urgent need to better understand the use of these practices and experiences of staff working with mental health consumers in inpatient settings and EDs.

This study reports the results of a survey of the perceptions and attitudes of nurses working with psychiatric consumers in Australia regarding the current use of seclusion and restraint, and their perceptions regarding elimination of such practices in inpatient psychiatric settings and EDs in Australia.

### Background

The agenda in Australia and other countries to reduce and eliminate seclusion and restraint is reflected in several key government and policy directives and clinical initiatives, particularly over the last decade. The National Mental Health Consumer & Carer Forum (2009) posits that seclusion and restraint are ‘not evidence‐based therapeutic interventions’, that they are ‘commonly associated with human rights abuse’, that they ‘cause short and long term emotional damage to consumers’, and that they ‘highlight a failure in care and treatment when they are used’ (p. 7). The Australian College of Mental Health Nurses (ACMHN) also published a Seclusion and Restraint Position Statement in 2016. This position statement sees restrictive practices (included also is chemical restraint) as last resort methods that should only be implemented with consideration of least restrictive care and implemented by trained mental health nurses and staff. The statement stresses the need to respect consumer dignity, engage in culturally appropriate care, meet consumer physical needs while they are secluded/restrained, and enact and discontinue practices with adherence to legal requirements. At a wider level, the policy statement stresses the need for research into alternatives to restrictive practice use and safe consumer management, as well as practice change (e.g. organizational culture, individual attitudes, leadership, staff training). Ultimately, it is the position of the ACMHN that seclusion and restraint use ‘be reduced and ultimately ended’ (Australian College of Mental Health Nurses, 2016, p. 4). Recently, the World Health Organization (2017) proposed QualityRights training initiatives on ending seclusion and restraint use. While seclusion and restraint are covered in less depth in the recently released Australian Fifth National Mental Health and Suicide Prevention Plan (Department of Health, 2017), seclusion is included as a practice to be addressed and monitored, and as one of the 24 key performance indicators under the domain of striving for ‘less avoidable harm’ in mental health care.

Evidence‐based initiatives, such as seclusion and restraint reduction programmes that use the Six Core Strategies for Reducing Seclusion and Restraint Use (Huckshorn 2004) and the Safewards model (Bowers 2014), have demonstrated positive effects. A systematic review of seclusion/restraint reduction programmes, most of which involved use of the six core strategies, concluded that ‘evidence argues in favor of programs that reduce SR use, without impacting the safety of health care providers’ (Goulet *et al*. 2017, p. 145), although which specific components were most effective was difficult to discern. In the case of Safewards, a U.K. cluster randomized controlled trial (Bowers *et al*. 2015) reported a 26.4% reduction in containment events; in Australia, a pre–post study reported a 36% reduction in seclusion following a roll‐out of the programme in Victoria (Fletcher *et al*. 2017). For such interventions, research is needed to evaluate whether reductions at study sites have been maintained, as well as whether substitute containment practices are used (see Noorthoorn *et al*. 2016). Despite the demonstrated efficacy of reduction initiatives, seclusion and restraint continue to be used worldwide with psychiatric consumers. For example, in a recent study of four European countries, Lepping *et al*. (2016) reported rates of between 4.5% (Southwest Germany) and 9.4% (the Netherlands) of consumers experiencing seclusion/restraint, with differences in rates according to the setting (e.g. forensic). Recently released national data of seclusion, physical restraint, and mechanical restraint in Australian public sector acute mental health hospital services for 2016–2017 revealed rates of 7.4, 8.3, and 0.9 events per 1000 bed days, respectively, with sometimes significant variations between states and territories (Australian Institute of Health and Welfare, 2018). For seclusion, there was a modest national reduction of 6.7% in events from 2012–2013 to 2016–2017.

While reduction is a positive step towards ensuring consumer‐focused care, health professionals demonstrate resistance to complete elimination of restraint and seclusion. In a large Australian study of health professionals, consumers, and carers, health professionals could identify the harms of seclusion and restraint. However, they were less likely than consumers or carers to believe it was desirable to eliminate the practices (Kinner *et al*. 2017). Similarly, in a qualitative study of staff and consumers’ views of restraint, an overarching theme involved restraint being seen as ‘a necessary evil’ (Wilson *et al*. 2017b, p. 503). Barriers to elimination in other qualitative studies included fear and perceptions of a lack of alternative methods to maintain safety; staff who were less experienced or lacked training in mental health; problematic staff–consumer relationships (e.g. not meeting or insensitive responding to consumer needs); and the physical environments of units (e.g. noise or lack of low‐stimulation spaces) not being conducive to reducing irritation and aggression (Muir‐Cochrane *et al*. 2015, 2018).

However, we know comparatively little at a wider level regarding the perceptions and attitudes of nurses towards containment practices, experiences of using the methods, thoughts regarding their elimination, and barriers but also enablers to elimination. Changes in consumer profiles such as increased acuity and, particularly in EDs, increases in presentations of substance‐affected consumers reflect an urgent need to investigate what factors drive attitudes towards seclusion and restraint reduction and elimination. This study was conducted to investigate specifically these factors.

## Method

### Design

The study involved the delivery of an online anonymous survey through the SurveyMonkey (SurveyMonkey Inc., San Mateo, CA, USA) platform to nurses working with psychiatric consumers to investigate their perceptions regarding the use of seclusion, physical restraint, and mechanical restraint. Definitions used in the survey were as follows: seclusion as the ‘deliberate confinement of the consumer alone in a room or area from which free exit is prevented’; physical restraint as ‘hands‐on immobilisation, holding the consumer or restriction of the consumer's freedom of movement by staff’; and mechanical restraint as ‘restricting a consumer's freedom of movement with devices such as jackets, belts, cuffs, and soft shackles’.

Respondents were recruited through the memberships of the Australian College of Mental Health Nurses (ACMHN), the Australian College of Nursing (ACN), and the Australian Nursing and Midwifery Federation (ANMF). Details of the research project were made available to members of these groups through their email distribution lists (for ACMHN and ACN members), websites, social media platforms (e.g. Twitter and Facebook pages), newsletters, and local branches (for ANMF members). Information provided to members consisted of a short description of the project and the URL to access the survey. Nurses working in an Australian psychiatric inpatient unit or ED were eligible to participate. The survey was available from 7 April to 25 May 2017. Ethical approval for the study was granted by the Flinders University Social and Behavioural Research Ethics Committee (approval number: 7588).

### Data collection

The survey comprised several sections examining respondent perceptions of the use of containment methods (seclusion, physical restraint, and mechanical restraint), more general workplace experiences, and demographic questions.

Individual items in the survey were either drawn from previously designed measures of attitudes to seclusion, restraint, and working practices with psychiatric consumers or specifically written for the project. As analysis largely involved examination of answers to individual items, modifications to existing measures and response scales were deemed appropriate. Items were completed using a 5‐point Likert‐type response scale, ranging from 1 (*strongly disagree*) to 5 (*strongly agree*), with the 5‐point Likert‐type response scale for items measuring the likelihood of seclusion/physical restraint/mechanical restraint use ranging from 1 (*Very unlikely S/PR/MR will be used*) to 5 (*Very likely S/PR/MR will be used*).

### Measures

#### Involvement in seclusion and restraint

Participants were asked (yes/no) if they had ever been involved in the use of (1) seclusion, (2) physical restraint, and (3) mechanical restraint.

#### General perceptions of containment

Perceptions of containment (i.e. not specific to any one of the three methods investigated) were examined using specifically written items based on the literature (Mann‐Poll *et al*. 2011, 2013; Wilson *et al*. 2017b) and items from/adapted from two measures: Staff Attitude to Coercion Scale (SACS; Husum *et al*. 2008), which measures nurses’ perceptions regarding seclusion and restraint use, including the extent to which these practices prevent dangerous situations, are necessary, and can be reduced; and the Seclusion and Restraint Experience Questionnaire (SREQ; Korkeila *et al*. 2016), which measures nurses’ emotions towards and experiences of use of seclusion/restraint, and perceptions of ethical/practical implications of their use. This section comprised 23 items.

#### Perceptions of specific containment methods

Specific perceptions of each containment method were examined separately using the seclusion, physical restraint, and mechanical restraint sections of the Attitudes to Containment Methods Questionnaire (Bowers *et al*., 2004). This measure examines nurses’ attitudes towards specific containment methods such as perceived efficacy, safety, and acceptability. For each containment method, respondents indicate their agreement with six items. Total scores for each section can range between 6 and 30, where higher scores indicate more positive attitudes towards the use of the specific containment method. Internal consistency reliabilities (Cronbach's alpha) in this study were all high: seclusion *α *= 0.91; physical restraint *α *= 0.88; mechanical restraint *α *= 0.92.

#### Use of seclusion and restraint in respondents’ workplaces and potential for elimination

Respondents were asked whether each containment method was used in their unit and, if so, to what extent they believed the method could be eliminated.

Experiences of seclusion/restraint use in workplaces, including perceptions regarding overuse, alternatives to minimize use, and reasons for use were measured using a total of 11 items, some from/adapted from the Seclusion and Restraint Experience Questionnaire (SREQ; Korkeila *et al*. 2016) and others developed based on literature review.

Researcher‐devised items based on literature review (Boumans *et al*. 2012; Mann‐Poll *et al*. 2011) and our own previous research (e.g. Oster *et al*. 2016) measured respondents’ perceptions regarding whether consumer behaviours and characteristics (e.g. aggression and violence; 17 items) and unit/staff factors (e.g. lack of adequate staffing, feeling inadequately skilled for duties; 38 items) made it more or less likely that seclusion and restraint would be used; for unit/staff factors, several items were from/adapted from the Mental Health Professionals Stress Scale (Cushway *et al*. 1996) and one item from the Essen Climate Evaluation Schema (Schalast *et al*. 2008).

#### Confidence in managing consumer aggression and potentially dangerous situations

As seclusion and restraint are containment methods used to manage potentially dangerous behaviour and maintain safety on a unit, respondents were asked about their confidence in working with aggressive consumers, practising de‐escalation, and maintaining safety on the unit. Nurses’ perceptions of safety in their workplace and confidence in unit procedures regarding managing aggression were measured using adapted items (and one additional item adapted from Schalast *et al*. 2008) from the 7‐item Confidence in Managing Inpatient Aggression Questionnaire (Martin & Daffern 2006), which measures confidence in dealing with consumers who are aggressive, maintaining safety, and using seclusion/restraint if needed.

### Data analysis

Data were analysed using IBM SPSS Statistics for Windows, version 23.0 (IBM Corp., Armonk, NY, USA). Of 533 complete responses, data were removed if respondents indicated they worked outside of Australia or solely in a service that was not an inpatient unit or emergency department. This resulted in the removal of 21 respondents. Data were then coded for subsequent statistical analysis.

Descriptive statistics (frequencies, means, standard deviations, medians, and ranges) are used to describe participant perceptions regarding the use of seclusion and restraint in inpatient and ED settings and to examine perceptions and attitudes regarding the potential for elimination. As only the Bowers *et al*. (2004) seclusion, physical restraint, and mechanical restraint measures were used in their entirety, examination of responses to individual items in each section of the survey is undertaken. This was seen to be more useful to understanding nurses’ attitudes rather than summing individual items into total scales and reporting only these total scores. For the Bowers *et al*. (2004) measures, total scores were calculated and one‐way repeated measures ANOVA was used to examine whether there were statistically significant differences in attitudes towards seclusion, physical restraint, and mechanical restraint. The Friedman test, a nonparametric test suitable for ordinal data, was used to determine whether there were statistically significant differences in respondents’ beliefs regarding the potential for elimination of seclusion, physical restraint, and mechanical restraint for respondents who worked in units where all three methods used. To assess the nature of these differences, Wilcoxon signed‐rank tests were performed. A Bonferroni correction was applied to the level of significance based on the number of comparisons (*P = *0.017). Wilcoxon signed‐rank tests were also used to examine differences in beliefs regarding elimination for respondents working in units using at least two of the methods. Effect sizes (*r*) were calculated to investigate the magnitude of observed effects, with *r = *0.10 indicating a small effect; *r = *0.30 a medium effect; and *r = *0.50 a large effect size (Cohen 1992; Field 2014).

## Results

### Survey respondent demographics

The sample consisted of 512 nurses, equivalent to approximately 2.46% of the mental health nursing workforce (Australian Institute of Health and Welfare, 2016). There were 368 female respondents (71.9%), 141 male respondents (27.5%), and three respondents who identified as ‘other’ (0.6%). Mean age (*n = *509) of respondents was 47.73 years (SD* = *11.54, *range* = 21–72 years).

Nearly 90% of the sample were registered nurses (RNs; *n = *460, 89.84%), with 72.17% (*n = *332) of these either having qualifications in mental health nursing (*n = *258) or being a credentialed mental health nurse (*n = *74). The remaining respondents were solely registered in mental health (*n = *30, 5.86% of the sample), enrolled nurses (*n = *17, 3.32%), or another type of nurse (*n = *5).

Over 60% of respondents (*n = *322, 62.89%) indicated that their highest level of education was completion of a postgraduate degree, the most common being a Master's degree (*n = *150), followed by other postgraduate qualifications such as a postgraduate diploma (*n = *164) and PhD/doctorate (*n = *8, 1.56%). Approximately 20% (*n = *109, 21.29%) of respondents indicated that their highest level of qualification was a Bachelor degree, with the remainder of the sample indicating another qualification such as hospital‐based training (*n = *34, 6.64%), diploma (*n = *23, 4.49%) or advanced diploma (*n = *14, 2.73%), and other qualifications (*n = *10, 1.95%).

Respondents were experienced clinicians, having worked in nursing practice for a median of 18 years, with their experience ranging from 3 months to 54 years (*n = *509). Seventy‐three (14.34%) had 5 years or less experience in nursing.

### Respondent unit details

All Australian states and territories were represented in the survey, with the largest numbers of respondents working in Queensland (*n = *127, 24.8%), followed by New South Wales (*n = *120, 23.4%), South Australia (*n = *103, 20.1%), Victoria (*n = *101, 19.7%), Western Australia (*n = *30, 5.86%), Tasmania (*n = *12, 2.34%), Australian Capital Territory (*n = *12, 2.34%), and Northern Territory (*n = *7, 1.37%).

Approximately 60% of respondents practised in a capital city (*n = *307, 59.96%) and 20.51% worked in a noncapital city metropolitan area (>100 000 population; *n = *105). A further 92 respondents worked in a rural area (17.97%), and four respondents each worked in a remote zone or reported an ‘other’ location.

Over 70% of respondents either worked in an acute adult psychiatric inpatient unit (*n = *257, 50.20%) or an emergency department (*n = *110, 21.48%). Table 1 presents current area of work of respondents. Respondents had worked in their current unit for a median of 5 years (*range = *3 weeks‐32 years; *n = *506).

**Table 1:** Respondents’ workplaces

Work area	*n* (%)
Acute adult psychiatric inpatient unit	257 (50.20%)
Emergency Department	110 (21.48%)
Child and adolescent psychiatric inpatient unit	31 (6.05%)
Forensic acute unit	22 (4.30%)
Psychiatric intensive care unit	15 (2.93%)
Forensic rehabilitation unit	15 (2.93%)
Older persons’ psychiatric inpatient/assessment unit	14 (2.73%)
High dependency psychiatric unit	11 (2.15%)
Rehabilitation psychiatric unit	9 (1.76%)
Intermediate care psychiatric unit	6 (1.17%)
Short‐stay psychiatric emergency unit/Clinical decision unit	5 (0.98%)
Emergency extended care psychiatric unit	3 (0.59%)
Mother and baby unit	3 (0.59%)
Older persons’ psychiatric rehabilitation unit	3 (0.59%)
Rural short‐stay acute psychiatric unit	2 (0.39%)
Secure extended care unit	2 (0.39%)
Eating disorders unit	1 (0.20%)
Other	3 (0.59%)

Respondents predominantly worked in a clinical role (*n = *411, 80.27%), with 63 (12.30%) working in management, 28 (5.47%) in education, four in administration (0.78%), and six (1.17%) in an ‘other’ role.

### Involvement in seclusion and restraint

Over 95% of respondents had been involved in the use of seclusion (95.31%, *n = *488) and physical restraint (96.48%, *n = *494), with less involvement in mechanical restraint (63.48%, *n = *325).

### General perceptions of containment

Respondents’ evaluations of all three containment methods as a whole (referred to as S/PR/MR) are presented in Table 2.

**Table 2:** General perceptions of containment methods

Item	Strongly disagree *n* (%)	Disagree *n* (%)	Neither disagree nor agree *n* (%)	Agree *n* (%)	Strongly agree *n* (%)	*M* (SD)
(1) All alternative methods should be tried before using S/PR/MR	12 (2.34%)	9 (1.76%)	13 (2.54%)	109 (21.29%)	369 (72.07%)	4.59 (0.83)
(2) Alternative methods cannot totally replace the use of S/PR/MR	30 (5.86%)	51 (9.96%)	60 (11.72%)	213 (41.6%)	158 (30.86%)	3.81 (1.15)
(3) I feel uncertain about how S/PR/MR affects the consumer	102 (19.92%)	235 (45.90%)	83 (16.21%)	75 (14.65%)	17 (3.32%)	2.36 (1.06)
(4) It is difficult to decide when to seclude or restrain	81 (15.82%)	246 (48.05%)	68 (13.28%)	96 (18.75%)	21 (4.10%)	2.47 (1.09)
(5) It is difficult to find alternative methods to S/PR/MR	82 (16.02%)	181 (35.35%)	82 (16.02%)	130 (25.39%)	37 (7.23%)	2.72 (1.21)
(6) S/PR/MR violates the autonomy of the consumer	22 (4.30%)	67 (13.09%)	110 (21.48%)	193 (37.70%)	120 (23.44%)	3.62 (1.10)
(7) Use of S/PR/MR is necessary as protection in dangerous situations	11 (2.15%)	22 (4.30%)	72 (14.06%)	199 (38.87)	208 (40.63%)	4.11 (0.95)
(8) For safety reasons S/PR/MR must sometimes be used	0 (0%)	25 (4.88%)	27 (5.27%)	228 (44.53%)	232 (45.31%)	4.30 (0.78)
(9) Use of S/PR/MR can harm the therapeutic relationship	13 (2.54%)	57 (11.13%)	59 (11.52%)	210 (41.02)	173 (33.79)	3.92 (1.06)
(10) Use of S/PR/MR is a declaration of failure on the part of the treating team	176 (34.38%)	180 (35.16%)	74 (14.45%)	52 (10.16%)	30 (5.86%)	2.18 (1.18)
(11) S/PR/MR may represent care and protection	27 (5.27%)	54 (10.55%)	86 (16.80%)	231 (45.12%)	114 (22.27%)	3.69 (1.09)
(12) More S/PR/MR should be used in the management of disturbed consumers	135 (26.37%)	181 (35.35%)	133 (25.98%)	33 (6.45%)	30 (5.86%)	2.30 (1.10)
(13) S/PR/MR may prevent the development of a dangerous situation	14 (2.73%)	60 (11.72%)	70 (13.67%)	256 (50.00%)	112 (21.88%)	3.77 (1.01)
(14) S/PR/MR violates the consumer's integrity	28 (5.47%)	93 (18.16%)	154 (30.08%)	166 (32.42%)	71 (31.87%)	3.31 (1.09)
(15) For severely ill consumers S/PR/MR may ensure safety	21 (4.10%)	33 (6.45%)	65 (12.70%)	258 (50.39%)	135 (26.37%)	3.8 (1.00)
(16) Use of S/PR/MR is necessary towards dangerous and aggressive consumers	20 (3.91%)	58 (11.33%)	118 (23.05%)	181 (35.35%)	135 (26.37%)	3.69 (1.10)
(17) Too much S/PR/MR is used in consumer care	58 (11.33%)	152 (29.69%)	140 (27.34%)	87 (16.99%)	75 (14.65%)	2.93 (1.23)
(18) Scarce resources lead to more use of S/PR/MR	28 (5.47%)	79 (15.43%)	67 (13.09%)	179 (34.96%)	159 (31.05%)	3.71 (1.21)
(19) Security guards are necessary in S/PR/MR	77 (15.04%)	128 (25.00%)	93 (18.16%)	108 (21.09%)	106 (20.70%)	3.07 (1.37)
(20) S/PR/MR could be reduced, given more time and personal contact with consumers	19 (3.71%)	62 (12.11%)	94 (18.36%)	163 (31.84%)	174 (33.98%)	3.80 (1.14)
(21) S/PR/MR should not be used at all	200 (39.06%)	153 (29.88%)	94 (18.36%)	36 (7.03%)	29 (5.66%)	2.10 (1.16)
(22) It will always be necessary to use S/PR/MR	37 (7.23%)	79 (15.43%)	159 (31.05%)	147 (28.71%)	90 (17.58%)	3.34 (1.15)
(23) Seclusion is a ‘necessary evil’	51 (9.96%)	74 (14.45%)	139 (27.15%)	174 (33.98%)	74 (14.45%)	3.29 (1.18)

Items 1–6 from/adapted from Korkeila *et al*. (2016); Items 7–18, 20 from/adapted from Husum *et al*. (2008).

Respondents expressed a need for the use of seclusion, physical restraint, and mechanical restraint. They believed that S/PR/MR use was necessary to maintain safety (Item 8) and protection (Item 7). While they strongly believed that S/PR/MR be used only after all alternative methods had been tried (Item 1) and that it was not difficult to find alternative methods (Item 4), a containment‐free environment was not strongly endorsed. Respondents moderately agreed that ‘it will always be necessary to use S/PR/MR’ (Item 22) with 46.29% of respondents indicating that they ‘agreed’ or ‘strongly agreed’ with this statement; 31.05% neither agreeing nor disagreeing with the statement; and 22.66% indicating that they ‘disagreed’ or ‘strongly disagreed’ with the need for continual use of containment.

Respondents were aware of the harms associated with containment method use, including potential damage to the therapeutic relationship (Item 9) and violation of consumer autonomy (Item 6). At the same time, respondents reported similar levels of agreement with other items that tapped perceptions that containment methods ‘may represent care and protection’ (Item 11) or prevent ‘the development of a dangerous situation’ (Item 13).

Respondents did not find it difficult to decide when to enact S/PR/MR (Item 4). In terms of underlying reasons for seclusion or restraint use, respondents tended to somewhat agree that scarce resources lead to increased use of S/PR/MR (Item 18) and that more time and personal contact with consumers could help reduce the use of these methods (Item 20).

### Perceptions of specific containment methods

Table 3 presents the means and standard deviations for the items measuring specific perceptions of seclusion, physical restraint, and mechanical restraint and their perceived safety, effectiveness, and acceptability. There was a significant effect of type of containment method on attitudes, *F*(1.86, 951.04)* = *153.30, *P < *0.001. Post hoc tests revealed that seclusion was perceived more favourably than physical restraint or mechanical restraint (both *P < *0.001) and that physical restraint was perceived more favourably than mechanical restraint (*P < *0.001). It should be noted, however, that mean scores for all methods were towards the mid‐range of possible scores, indicating mixed perceptions of seclusion, physical restraint, and mechanical restraint.

**Table 3:** Perceptions of specific containment methods (Attitudes to Containment Methods Questionnaire; Bowers *et al*. 2004)

Item	Seclusion *M* (SD)	Physical restraint *M* (SD)	Mechanical restraint *M* (SD)
(1) … respects consumers’ dignity	2.73 (1.19)	2.42 (1.05)	1.98 (0.98)
(2) … is safe for the staff who use it	2.98 (1.56)	2.41 (1.07)	2.80 (1.15)
(3) … is safe for the consumer who is subject to it	3.03 (1.62)	2.55 (1.08)	2.57 (1.67)
(4) Overall, … is acceptable	3.27 (1.16)	3.13 (1.12)	2.53 (1.18)
(5) Overall, …is effective	3.35 (1.08)	3.24 (1.06)	2.80 (1.18)
(6) I would be prepared to use …	3.87 (1.00)	3.68 (1.01)	2.85 (1.27)
Total M (SD)	19.24 (5.60)	17.42 (5.08)	15.55 (5.89)

There was adaptation of item wording for consistency with other parts of the survey.

### Seclusion and restraint in respondents’ workplaces and potential for elimination

Most respondents indicated that physical restraint was used in their unit (*n = *474, 92.58%), followed by seclusion (*n = *422, 82.42%). Fewer (*n = *195, 38.09%) reported that mechanical restraint was used in their unit. Respondents differed in how many methods were used on their individual units, with 144 (28.13%) indicating that *all three methods* were used while 19 (3.71%) indicated that *none* of the methods were used on their unit. Table 4 presents the different combinations of use of the three methods. Examining the settings where the most respondents worked, over 90% of respondents from acute adult psychiatric inpatient units reported that their units used seclusion (*n = *239, 93.00%) and physical restraint (*n = *238, 92.61%). Over 95% of respondents from EDs reported use of physical restraint (*n = *105, 95.45%), with almost 60% (*n = *64, 58.18%) reporting the use of seclusion. Over 75% of ED respondents (*n = *84, 76.36%), but less than 30% (*n = *75, 29.18%) of acute adult psychiatric inpatient unit respondents reported use of mechanical restraint. Of 31 respondents from a child and adolescent psychiatric inpatient unit, physical restraint (*n = *30) and seclusion (*n = *29) were reported to be used in their units, but rarely mechanical restraint (*n = *1). Of 22 respondents who worked in forensic acute units, 21 indicated seclusion use, 20 the use of physical restraint, and 8 the use of mechanical restraint.

**Table 4:** Types of containment methods used at specific workplace unit

Type(s) of containment used	*n*
Seclusion and physical restraint	260 (50.78%)
Seclusion, physical restraint, mechanical restraint	144 (28.13%)
Physical and mechanical restraint	49 (9.57%)
Physical restraint only	21 (4.10%)
Seclusion only	17 (3.32%)
Seclusion and mechanical restraint	1 (0.20%)
Mechanical restraint only	1 (0.20%)
None	19 (3.71%)

Respondents who indicated that a method was used in their workplace largely disagreed that the method could be eliminated, with the elimination of mechanical restraint seen as more of a possibility (*M = *2.43, SD* = *1.28) than seclusion (*M = *2.31, SD* = *1.25) and physical restraint (*M = *2.12, SD* = *1.11).

For respondents who reported that all three methods were used on their units, there was a statistically significant difference in beliefs regarding elimination depending on containment method, *χ* ^2^(144)* = *37.70, *P < *0.001. Respondents were significantly *more likely* to agree that mechanical restraint (*M = *2.51, SD* = *1.31) could be eliminated compared to either seclusion (*M = *2.08, SD* = *1.24), *T = *441, *P < *0.001, *r = *−0.26, or physical restraint (*M = *2.01, SD* = *1.18), *T = *218.50, *P < *0.001, *r = *−0.28. There was no statistically significant difference in beliefs regarding elimination of seclusion versus physical restraint.

There were statistically significant differences in beliefs regarding potential for elimination for those respondents who indicated seclusion and physical restraint (but not mechanical restraint) were used on their units, *T = *592.50, *P < *0.001, *r = *‐0.20. In this case, respondents were more likely to believe that seclusion (*M = *2.45, SD* = *1.25) rather than physical restraint (*M = *2.20, SD* = *1.08) could be eliminated from their units, Finally, there were significant differences between beliefs in elimination for respondents working in units that used physical and mechanical restraint (but not seclusion), *T = *13.50, *P < *0.01, *r = *−0.27, with greater agreement that mechanical restraint (*M = *2.20, SD* = *1.15) rather than physical restraint (*M = *1.80, SD* = *0.76) could be eliminated from respondent units.

Table 5 presents respondents’ perceptions of containment methods at their specific unit or ED (*n = *493, excluding those who indicated that *no* containment methods were used in their service). Respondents tended to disagree that S/PR/MR was used too often (Item 1) or that alternative methods were not sufficiently employed to minimize S/PR/MR use (Item 2). They also were more likely to disagree that there were conflicts between attempts to eliminate S/PR/MR and organizational policy (Item 5), or that practice on their units was at odds with guidelines related to S/PR/MR practice (Item 4).

**Table 5:** Perceptions of use of containment methods at specific workplace unit

Item	Strongly disagree *n* (%)	Disagree *n* (%)	Neither disagree nor agree *n* (%)	Agree *n* (%)	Strongly agree *n* (%)	*M* (SD)
(1) S/PR/MR are used too often in my unit	114 (23.12%)	197 (39.96%)	67 (13.59%)	74 (15.01%)	41 (8.32%)	2.45 (1.23)
(2) Alternatives to minimize the use of S/PR/MR have not been used as much as possible in my unit	92 (18.66%)	174 (35.29%)	54 (10.95%)	109 (22.11%)	64 (12.98%)	2.75 (1.34)
(3) There are different opinions about the need to use S/PR/MR in my unit	26 (5.27%)	71 (14.40%)	67 (13.59%)	236 (47.87%)	93 (18.86%)	3.61 (1.11)
(4) The guidelines related to S/PR/MR practices are not followed in my unit	127 (25.76%)	209 (42.39%)	68 (13.79%)	65 (13.18%)	24 (4.87%)	2.29 (1.13)
(5) Organisational policy conflicts with attempts to eliminate S/PR/MR in my unit	70 (14.20%)	186 (37.73%)	127 (25.76%)	86 (17.44%)	24 (4.87%)	2.61 (1.08)
(6) Some nurses in my unit are more willing to use S/PR/MR than others	24 (4.87%)	60 (12.17%)	64 (12.98%)	227 (46.04%)	118 (23.94%)	3.72 (1.10)
(7) I feel pressure to use S/PR/MR in my unit	150 (30.43%)	202 (40.97%)	64 (12.98%)	60 (12.17%)	17 (3.45%)	2.17 (1.10)
(8) I have misgivings regarding S/PR/MR use in my unit	92 (18.66%)	185 (37.53%)	93 (18.86%)	88 (17.85%)	35 (7.10%)	2.57 (1.18)
(9) I don't question the use of S/PR/MR in my unit	118 (23.94%)	247 (50.10%)	72 (14.60%)	37 (7.51%)	19 (3.85%)	2.17 (1.00)
(10) S/PR/MR can't be reduced without compromising safety in my unit	66 (13.39%)	159 (32.25%)	99 (20.08%)	94 (19.07%)	75 (15.21%)	2.90 (1.29)
(11) S/PR/MR can be reduced in my unit	35 (7.10%)	104 (21.10%)	124 (25.15%)	147 (29.82%)	83 (16.84%)	3.28 (1.18)

Items 1–4, 6 from/adapted from Korkeila *et al*. (2016).

Respondents indicated that there were differences in opinion between unit staff regarding the use of S/PR/MR (Item 3) and willingness to use containment methods (Item 6). However, they did not have strong misgivings about the use of S/PR/MR on their unit (Item 8) or feel pressure to use S/PR/MR (Item 7).

### Perceived consumer behaviours and unit factors influencing seclusion and restraint use

Respondents were asked to consider consumer behavioural factors (Table 6) and unit factors (Table 7) that they believed to increase or decrease the likelihood that containment measures will be used in their individual unit. The behaviours considered most likely to be involved in seclusion or restraint were actual physical aggression and violence, with 54.16% of respondents indicating that it is ‘very likely S/PR/MR will be used’. Other behaviours that made it more likely for seclusion and restraint as intervention strategies were damage to property and consumers being intoxicated (alcohol or drugs). Respondents also believed previous seclusion or restraint could predict current S/PR/MR use.

**Table 6:** Consumer behavioural factors influencing the use of containment methods

Item	Very unlikely S/PR/MR will be used *n* (%)	Unlikely S/PR/MR will be used *n* (%)	Neither unlikely nor likely *n* (%)	Likely S/PR/MR will be used *n* (%)	Very likely S/PR/MR will be used *n* (%)	*M* (SD)
(1) Verbal aggression	224 (45.44%)	162 (32.86%)	62 (12.58%)	35 (7.10%)	10 (2.03%)	1.87 (1.02)
(2) Threats of physical aggression	53 (10.75%)	140 (28.40%)	112 (22.72%)	158 (32.05%)	30 (6.09%)	2.94 (1.13)
(3) Actual physical aggression/violence	0 (0%)	13 (2.64%)	23 (4.67%)	190 (38.54%)	267 (54.16%)	4.44 (0.71)
(4) Absconding (attempts or actual)	131 (26.57%)	134 (27.18%)	93 (18.86%)	108 (21.91%)	27 (5.48%)	2.53 (1.25)
(5) Intrusive behaviour	145 (29.41%)	189 (38.34%)	90 (18.26%)	65 (13.18%)	4 (0.81%)	2.18 (1.02)
(6) Attempted suicide and/or self‐harm	122 (24.75%)	129 (26.17%)	94 (19.07%)	100 (20.28%)	48 (9.74%)	2.64 (1.31)
(7) Damage to property	48 (9.74%)	90 (18.26%)	115 (23.33%)	173 (35.09%)	67 (13.59%)	3.25 (1.19)
(8) Disruptive behaviour	95 (19.27%)	170 (34.48%)	104 (21.10%)	108 (21.91%)	16 (3.25%)	2.55 (1.13)
(9) Impulsive behaviour	89 (18.05%)	163 (33.06%)	153 (31.03%)	78 (15.82%)	10 (2.03%)	2.51 (1.03)
(10) Agitation	92 (18.66%)	185 (37.73%)	117 (23.73%)	83 (16.84%)	16 (3.25%)	2.48 (1.08)
(11) Disorientation	176 (35.70%)	199 (40.37%)	82 (16.63%)	32 (6.49%)	4 (0.81%)	1.96 (0.93)
(12) Consumer is intoxicated (alcohol and/or drugs)	98 (19.88%)	116 (23.53%)	143 (29.01%)	86 (17.44%)	50 (10.14%)	2.74 (1.24)
(13) Consumer is withdrawing from alcohol or methamphetamines	116 (23.53%)	137 (27.79%)	120 (24.34%)	81 (16.43%)	39 (7.91%)	2.57 (1.23)
(14) Consumer is new to the unit	220 (44.62%)	149 (30.22%)	102 (20.69%)	44 (4.46%)	0 (0%)	1.85 (0.90)
(15) Consumer is under an involuntary admission order	165 (33.47%)	132 (26.77%)	117 (23.73%)	64 (12.98%)	15 (3.04%)	2.25 (1.14)
(16) Consumer has previously been secluded or physically/mechanically restrained	99 (20.08%)	118 (23.94%)	156 (31.64%)	97 (19.68%)	23 (4.67%)	2.65 (1.14)
(17) Staff cannot communicate effectively with the consumer	127 (25.76%)	135 (27.38%)	124 (25.15%)	79 (16.02%)	29 (5.68%)	2.48 (1.20)

**Table 7:** Unit factors influencing the use of containment methods

Item	Very unlikely S/PR/MR will be used *n* (%)	Unlikely S/PR/MR will be used *n* (%)	Neither unlikely nor likely *n* (%)	Likely S/PR/MR will be used *n* (%)	Very likely S/PR/MR will be used *n* (%)	*M* (SD)
(1) Lack of support from management	76 (1615.42%)	92 (18.66%)	162 (32.86%)	118 (23.94%)	45 (9.13%)	2.93 (1.19)
(2) Conflict with other professionals	89 (18.05%)	118 (23.94%)	173 (35.09%)	89 (18.05%)	24 (4.87%)	2.68 (1.11)
(3) Lack of adequate staffing	47 (9.53%)	64 (12.98%)	100 (20.28%)	208 (42.19%)	74 (15.01%)	3.40 (1.17)
(4) Lack of trust/confidence in colleagues	68 (13.79%)	101 (20.49%)	157 (31.85%)	125 (25.35%)	42 (8.52%)	2.94 (1.16)
(5) Feeling inadequately skilled for dealing with emotional needs of consumers	80 (16.23%)	107 (21.70%)	128 (25.96%)	130 (26.37%)	48 (9.74%)	2.92 (1.23)
(6) Conflicting roles with other professionals	93 (18.86%)	117 (23.73%)	174 (35.29%)	86 (17.44%)	23 (4.67%)	2.65 (1.11)
(7) Uncertainty about own capabilities	95 (19.27%)	132 (26.77%)	147 (29.82%)	93 (18.86%)	26 (5.27%)	2.64 (1.15)
(8) Not enough time to complete all tasks satisfactory	112 (22.72%)	115 (23.33%)	138 (27.99%)	93 (18.86%)	35 (7.10%)	2.64 (1.22)
(9) Lack of good staff role models	68 (13.79%)	72 (14.60%)	125 (25.35%)	160 (32.45%)	68 (13.79%)	3.18 (1.24)
(10) Feeling inadequately skilled for working with acutely ill consumers	75 (15.21%)	87 (17.65%)	116 (23.53%)	162 (32.86%)	53 (10.75%)	3.06 (1.24)
(11) Too many consumers on the unit	80 (16.23%)	84 (17.04%)	128 (25.96%)	138 (27.99%)	63 (12.78%)	3.04 (1.27)
(12) Poor management or supervision	62 (12.58%)	78 (15.82%)	136 (27.59%)	160 (32.45%)	57 (11.56%)	3.15 (1.20)
(13) Lack of clinical supervision	76 (15.42%)	91 (18.46%)	148 (30.02%)	127 (25.76%)	51 (10.34%)	2.97 (1.21)
(14) Lack of adequate staff in a potentially dangerous environment	38 (7.71%)	41 (8.32%)	86 (17.44%)	197 (39.96%)	131 (26.57%)	3.69 (1.17)
(15) Working long hours/shifts	92 (18.66%)	93 (18.86%)	179 (36.31%)	86 (17.44%)	43 (8.72%)	2.79 (1.19)
(16) Presence of security guards in the unit	112 (22.72%)	127 (25.76%)	155 (31.44%)	70 (14.20%)	29 (5.88%)	2.55 (1.16)
(17) Poor physical environment	71 (14.40%)	66 (13.39%)	128 (25.96%)	169 (34.28%)	59 (11.97%)	3.16 (1.23)
(18) Noise in the unit	85 (17.24%)	95 (19.27%)	138 (27.99%)	148 (30.02%)	27 (5.48%)	2.87 (1.18)
(19) Lack of guards in the unit	109 (22.11%)	131 (26.57%)	162 (32.86%)	69 (14.00%)	22 (4.46%)	2.52 (1.11)
(20) Overcrowding in the unit	93 (18.86%)	82 (16.63%)	128 (25.96%)	150 (30.43%)	40 (8.11%)	2.92 (1.24)
(21) Lack of privacy in the unit	95 (19.27%)	108 (21.91%)	168 (34.08%)	94 (19.07%)	28 (5.68%)	2.70 (1.15)
(22) Staff fear of consumers	55 (11.16%)	63 (12.78%)	96 (19.47%)	189 (38.34%)	90 (18.26%)	3.40 (1.24)
(23) Too many rules on the unit	89 (18.05%)	108 (21.91%)	172 (34.89%)	94 (19.07%)	30 (6.09%)	2.73 (1.14)
(24) Formal training in S/PR/MR use	96 (19.47%)	172 (34.89%)	165 (33.47%)	41 (8.32%)	19 (3.85%)	2.42 (1.02)
(25) Positive ward/unit culture	155 (31.44%)	205 (41.58%)	106 (21.50%)	21 (4.26%)	6 (1.22%)	2.02 (0.90)
(26) Being able to build rapport with the consumer	213 (43.20%)	207 (41.99%)	57 (11.56%)	16 (3.25%)	0 (0%)	1.75 (0.78)
(27) Good communication and flow of information at work	187 (37.93%)	185 (37.53%)	100 (20.28%)	21 (4.26%)	0 (0%)	1.91 (0.86)
(28) Multidisciplinary team works well together	172 (34.89%)	207 (41.99%)	95 (19.27%)	19 (3.85%)	0 (0%)	1.92 (0.83)
(29) Good communication between staff	173 (35.09%)	204 (41.38%)	98 (19.88%)	18 (3.65%)	0 (0%)	1.92 (0.85)
(30) Taking the consumer's perspective and experiencing empathy	183 (37.12%)	181 (36.71%)	113 (22.92%)	16 (3.25%)	0 (0%)	1.92 (0.85)
(31) Compassion toward the consumer	187 (37.93%)	172 (34.89%)	120 (24.34%)	14 (2.84%)	0 (0%)	1.92 (0.86)
(32) Emotional support from colleagues	155 (31.44%)	179 (36.31%)	138 (27.99%)	21 (4.26%)	0 (0%)	2.05 (0.87)
(33) Keeping professional/clinical skills up to date	159 (32.25%)	188 (38.13%)	124 (25.15%)	22 (4.46%)	0 (0%)	2.02 (0.87)
(34) Clear organisational structure and policies	143 (29.01%)	178 (36.11%)	148 (30.02%)	20 (4.06%)	4 (0.81%)	2.12 (0.90)
(35) Using a trauma‐informed approach to the consumer	173 (35.09%)	182 (36.92%)	119 (24.14%)	19 (3.85%)	0 (0%)	1.97 (0.86)
(36) Taking a recovery‐oriented approach to the consumer	168 (34.08%)	164 (33.27%)	140 (28.40%)	15 (3.04%)	6 (1.22%)	2.04 (0.93)
(37) Staff who know consumers and their personal histories well	186 (37.73%)	200 (40.57%)	83 (16.84%)	24 (4.87%)	0 (0%)	1.89 (0.85)
(38) Having/using an individualized consumer care plan	151 (30.63%)	185 (37.53%)	139 (28.19%)	18 (3.65%)	0 (0%)	2.05 (0.86)

Items 1–3, 5–8, 10–12, 15, 17, 27–28, 32–34 from/adapted from Cushway et al. Tyler (1996); Item 37 adapted from Schalast *et al*. (2008).

In contrast to physical aggression, respondents thought it very unlikely that verbal aggression would result in seclusion or restraint use, with 45.44% of respondents believing it was ‘very unlikely S/PR/MR will be used’. Respondents also believed that disorientation (*M = *1.96, SD* = *0.93) or consumers being new to the unit (*M = *1.85, SD* = *0.90) were unlikely to lead to S/PR/MR use.

For unit factors, respondents believed that it was more likely that S/PR/MR would occur in units with lack of adequate staffing (Items 3 and 14), lack of good staff role models (Item 9), poor management or supervision (Item 12), poor physical environment (Item 17), and when there were too many consumers on the unit (Item 11). At an individual level, feeling inadequately skilled for working with acutely ill consumers was seen to make it more likely containment would be used (Item 10).

Factors that were seen to make it unlikely that S/PR/MR would be used were those that stressed nurse–consumer rapport (Item 26), knowing consumers’ histories well (Item 37), staff communicating and working well together (Items 27–29), empathy for consumers (Items 30–31), and using trauma‐informed care principles (Item 35).

### Confidence in managing consumer aggression and potentially dangerous situations

Table 8 presents respondents' perceptions of safety and confidence in managing aggression on their units. Respondents indicated that there was potential for threatening situations to occur on their unit (Item 7). Despite this, respondents were confident in their abilities to handle consumer aggression or hostility (Item 1). When specifically asked about the use of containment methods, nearly 85% (*n = *435, 84.96%) ‘agreed’ or ‘strongly agreed’ with the statement ‘I am able to contribute to the seclusion or restraint of an aggressive consumer’ (Item 4). Overall, respondents indicated that they felt moderately safe in their workplaces, although 21.88% of respondents ‘strongly disagreed’ or ‘disagreed’ that they felt safe. Respondents were also somewhat more confident in their own abilities to maintain safety (Item 5) than those of their colleagues (Item 6).

**Table 8:** Respondent confidence in managing consumer aggression and maintaining safety

Item	Strongly disagree *n* (%)	Disagree *n* (%)	Neither disagree nor agree *n* (%)	Agree *n* (%)	Strongly agree *n* (%)	*M* (SD)
(1) I am confident in my ability to work with hostile or aggressive consumers	0 (0%)	34 (6.64%)	40 (7.81%)	279 (54.49%)	159 (31.05%)	4.10 (0.80)
(2) I feel safe around aggressive consumers	35 (6.84%)	125 (24.41%)	133 (25.98%)	168 (32.81%)	51 (9.96%)	3.15 (1.11)
(3) I am able de‐escalate an aggressive consumer	0 (0%)	13 (2.54%)	91 (17.77%)	298 (58.20%)	110 (21.48%)	3.99 (0.70)
(4) I am able to contribute to the seclusion or restraint of an aggressive consumer	0 (0%)	28 (5.47%)	49 (9.57%)	302 (58.98%)	133 (25.98%)	4.05 (0.76)
(5) I am able to maintain my own safety in the presence of an aggressive consumer	0 (0%)	27 (5.27%)	74 (14.45%)	300 (58.59%)	111 (21.68%)	3.97 (0.76)
(6) I am confident in my colleagues’ ability to maintain safety and manage an aggressive consumer	20 (3.91%)	116 (22.66%)	147 (28.71%)	176 (34.38%)	53 (10.35%)	3.24 (1.04)
(7) Really threatening situations can occur in my unit	0 (0%)	13 (2.54%)	15 (2.93%)	157 (30.66%)	327 (63.87%)	4.56 (0.68)
(8) I feel safe in my unit	30 (5.86%)	82 (16.02%)	139 (27.15%)	200 (39.06%)	61 (11.91%)	3.35 (1.07)

Items 1‐6, 8 from/adapted from Martin and Daffern (2006); Item 7 adapted from Schalast *et al*. (2008).

## Discussion

This is the largest study of its size to date in Australia on nurses’ perceptions and experiences of seclusion and restraint. Overall, respondents believed that complete elimination of seclusion, physical restraint, and mechanical restraint were not possible. However, respondents identified a number of factors that were likely to help or hinder efforts to reduce, and, where possible, eliminate seclusion and restraint use.

Findings demonstrate that most nurses had been involved in seclusion, physical restraint, and, to a lesser degree, mechanical restraint, confirming existing clinical practice with mental health consumers. The necessity of restraint was supported in the context of dangerous situations, albeit as a last resort to protect consumers and staff (Kinner *et al*. 2017). Of interest is the spread of opinion and ambivalence regarding whether the use of containment methods will always be necessary, with 31.05% of respondents unsure about the need for continued containment methods use, 22.66% disagreeing with their continued use, and 46.29% agreeing that they will always be necessary. This spread in perceptions may be associated with the availability of appropriate less restrictive alternatives and deserves further examination as to reasons (Muir‐Cochrane *et al*. 2015). Respondents accepted that seclusion and restraint use were deleterious to their relationships with consumers, as other research has supported (Mohr *et al*. 2003). They also felt that containment use was a function of a lack of resources and could be reduced with more consumer contact. These findings add to the body of research identifying that nurses struggle with the dichotomy between care and control, but see safety as the primary motivation for use of restrictive methods (Riahi *et al*. 2016). It is important to note, however, that the systematic review by Goulet *et al*. (2017) found that ‘aggression and injury rates do not increase following implementation of an SR reduction program’ (p. 145).

Findings here reveal that nurses do not have difficulty in making decisions about the use of containment methods. Furthermore, nurses in this study do not perceive the use of containment as a failure on the part of the treating team, which is an important finding given critiques from some stakeholders who see restraint in such a light (Melbourne Social Equity Institute 2014). However, feeling not sufficiently skilled in caring for acutely ill consumers was seen to increase the likelihood of the use of containment measures, and so it is important to consider whether lack of difficulty in deciding to use containment is driven by a decision regarding this being the most appropriate intervention, or whether further training is needed in managing conflict and utilizing alternatives.

Most nurses indicated that physical restraint and seclusion were used in their units, with mechanical restraint less commonly adopted. Nurses perceived seclusion and physical restraint to be more effective, dignified for consumers, and acceptable than mechanical restraint, although seclusion was seen to be the most favourable form of containment. This may be due to the possibility of staff and consumer injuries during physical restraint and the ageing workforce of mental health nurses. It may also relate to perceptions regarding the most suitable of three methods, all for which there was moderate but not strong acceptance. While previous research suggests that mechanical restraint is amongst containment methods with the least approval by psychiatric inpatients and staff, different types of observation (e.g. intermittent observation), transfer/placement in another area (e.g. PICU transfer, timeout), or PRN medication are considered more favourable than seclusion or physical restraint (Whittington *et al*. 2009). Nurses did not believe that seclusion, physical restraint, or mechanical restraint could be eliminated, but had more support for the elimination of mechanical restraint. This is, perhaps, not surprising as mechanical restraint was used least in respondents’ units. Nurses did not feel containment methods were used excessively in their own units or that such use was outside of organizational policy, stating that alternative methods were used as much as possible. However, information was not collected as to the nature of these alternative methods or if this finding reflects respondents’ perceptions that seclusion and restraint were used when other methods had not resolved risk of harm (i.e. a last resort). Other research indicates that perceptions of the lack of availability of alternative methods influence reluctance to stop using seclusion and restraint, with a ‘dichotomy’ apparent between recommendations in reports/policy and clinical practice (Muir‐Cochrane *et al*. 2015, p. 113).

Respondents believed physical aggression and violence, consumer intoxication, and damage to property would increase the likelihood of the use of seclusion and restraint, which is consistent with both organizational policy and the current literature (Oster *et al*. 2016). Regarding substance use, health professionals working in ED departments with crystal methamphetamine (ICE) users describe their care as ‘challenging; at times distressing, and highly complex’ and that that their care is ‘resource‐intensive and the unpredictable behaviours that accompany ICE use meant that multiple staff were often needed’ (Cleary *et al*. 2017, p. 35). Mental health assessment to determine whether there are mental health issues warranting admission to a mental health facility was also seen to be problematic while the consumer was intoxicated. This highlights some of the complexity regarding the use of seclusion and restraint with these consumers.

The findings demonstrate that nurses did not believe that seclusion and restraint were likely to be used for consumers new to the unit. This contrasts with the literature, with seclusion/restraint use and other incidents, such as consumers under inpatient treatment orders leaving units without permission, occurring early in admission (Bullock *et al*. 2014; Gerace *et al*. 2015). It is important to note that except for cases of aggression and violence, nurses did not strongly believe that many consumer factors would likely lead to containment use. The reasons for these perspectives are unclear but may be a function of nurses in the study presenting an ‘ideal’ answer rather than what happens in practice.

Staff and unit factors influencing containment have received sustained attention in the literature (Pollard *et al*. 2007), and this study cites lack of good (staff) role models, inadequately trained staff, overcrowded units, and lack of management and supervision as key issues. Conversely, nurse–consumer engagement, effective communication, and trauma‐informed care approaches were seen to be facilitators to a least restrictive environment, also consistent with recent work (Gaynes *et al*. 2016). Indeed, these findings are reflected in reduction initiatives, with both strong leadership and workforce development, the latter stressing staff education and the fostering of recovery and trauma‐informed care, identified as two of the six core strategies for reducing seclusion and restraint use (Huckshorn, 2004; National Mental Health Commission 2015).

While nurses felt somewhat safe at work, they believed that threatening situations on their units were common, with 21.88% of respondents feeling unsafe at work and 31.25% feeling unsafe around aggressive consumers. These are important findings for stakeholders to consider in attempts to reduce containment. Feelings of lack of safety are unlikely to be conducive to least restrictive and quality de‐escalation processes of care. Furthermore, staff were more confident in their own abilities than those of their colleagues. This may suggest that educational preparation is not uniform across nursing groups or disciplines regarding containment method (and alternate methods) use. However, this may reflect disparities in judgements of one's own practice and actual practice. This should also be considered along with the finding that respondents felt that their colleagues differed in beliefs regarding the need for containment practices and willingness to use the methods. In a study of perceptions of mental illness, Reavley and Jorm (20112011) explained beliefs that one's personal attitudes towards mental illness differ from public perceptions with reference to the social psychological concept of pluralistic ignorance, ‘where most people erroneously perceive that they have different attitudes to the majority’ (p. 1092). This may result in colleagues who actually have similar private attitudes towards containment (e.g. reluctance to use) believing that others hold more favourable views, resulting in acceptance of containment becoming the norm on the unit (see Prentice & Miller 1996).

Unit culture factors can be usefully examined using the recent work of Bowers *et al*. (2017), where it was demonstrated that wards without seclusion were less likely to use manual (physical) restraint, indicating a cultural unit effect regarding perceptions of containment. However, units without designated seclusion rooms used more rapid tranquillization and used a side room to contain consumers. Hence, there is no evidence to date that removing seclusion rooms results in overall reductions in containment, but that substitute containment occurs. Evidence for substitute containment is seen in work by Noorthoorn *et al*. (2016), where seclusion was decreased but forced medication increased. This is significant in any consideration of elimination of seclusion rooms so that changes do not merely result in changing one form of restraint for another, potentially equally unpalatable one. Studies of consumer preferences for particular coercive interventions if deemed necessary are mixed, where less invasive procedures such as one‐to‐one observation are seen as preferable to seclusion, physical, or mechanical restraint (Krieger *et al*. 2018). However, comparisons between methods such as seclusion and forced medication indicate that while individual consumers may prefer one to the other, they identify significant negative impacts of the use of either method (Veltkamp *et al*. 2008).

This present study identified perceived facilitators of containment elimination involving trauma‐informed care principles, empathic nurse–consumer interaction, and collaborative staff relationships. Indeed, empathy involving perspective taking and concern has been identified as a means to defuse conflict between staff and consumers (Gerace *et al*. 2018), with unit conflict linked to the use of containment methods (Bowers 2014). Within the six core strategies and other interventions for preventing containment use, safety plans are included as a potential way to prevent distress and promote self‐control and the use of individualized de‐escalation strategies (Huckshorn 2004; Lewis *et al*. 2009). Such plans incorporate consumer preferences and take account of experiences such as previous trauma (Krieger *et al*. 2018). Safety plans have been demonstrated as effective in reducing the use of seclusion and restraint (Lewis *et al*. 2009), and in a Delphi study, experts identified the need for further research into patient‐centred approaches and consumer‐driven safety planning (Dewa *et al*. 2018). Within Australian contexts, researchers have similarly identified the need for research into trauma‐informed care in inpatient settings (Wilson *et al*. 2017a), and this seems a promising avenue to promoting alternative strategies to seclusion and restraint.

### Limitations

The sample was large, but does represent a small proportion of nurses working in mental health. There were also smaller numbers of respondents in units other than acute adult and emergency departments, as well as fewer respondents from rural and remote areas. It is possible that nurses who chose to participate differ from those who viewed study information and declined participation, and participation depended on nurses seeing the study listed through the professional organizations used for recruitment. Future studies could utilize ‘champions’ within health services to promote the study. However, this should be used carefully to avoid respondent perceptions of coercion to participate. Respondents were relatively more experienced in nursing. While the respondent group can be deemed representative of the national population of mental health nurses in terms of sex, age, qualification, work role, and geographical location (Australian Institute of Health and Welfare, 2016), the research indicates that age and experience are important to understanding staff members’ attitudes to use of specific methods of containment (Whittington *et al*. 2009). Examining the perceptions of the younger members of the workforce is, therefore, important to understand reduction efforts moving forward.

In the present study, it was also not possible to obtain actual benchmarks such as rates of seclusion/restraint in a respondent’s unit to compare to their perceptions of overuse, effectiveness, and so on. Respondents were not asked to indicate how recently they had used containment methods, or whether they had received any recent education (undergraduate or continuing education) about alternatives to containment use. While the anonymous nature of the survey reduces the risk of social desirability bias, it is possible that with a sensitive topic such as the use of seclusion and restraint, respondents may report attitudes they perceive to be more acceptable. Finally, other containment methods, such as chemical restraint, as well as the nature of the use of de‐escalation or availability of other strategies (e.g. sensory approaches, environmental modifications to units) in individual workplaces, need to be considered.

## Conclusion

In spite of calls for the reduction and elimination of seclusion, physical restraint, and mechanical restraint reflected at the policy or research level, these practices are still used in Australia and nurses hold mixed beliefs regarding their elimination. Nurses do not necessarily see the practices as favourable, but necessary for maintaining a safe work environment. Unless factors that have been identified as making elimination or at least significant reduction possible, such as those reflected in the six core strategies (Huckshorn 2004), are implemented at an organizational level, and nurses are provided with what they consider viable alternatives to their use, reduction, and, indeed, elimination are likely to be very problematic. This survey provides a large snapshot of nurses’ perceptions of containment use and seclusion and restraint practices in Australia. In this way, the survey provides data to inform practice, which has been identified as a necessity to containment reduction and elimination efforts (Mann‐Poll *et al*. 2015).

## Relevance for clinical practice

The focus of any seclusion/restraint reduction and elimination efforts should be not only on removing barriers that perpetuate their use, but also on enablers towards containment reduction and, where possible, elimination. At a wider level, the present findings highlight the importance to seclusion and restraint reduction and elimination efforts of strong clinical leadership, sufficient staff numbers and resources, consideration of the appropriateness of the physical unit environment, and appropriate resources for the use of alternative methods to seclusion and restraint that maintain staff and consumer safety. In addition, a focus on trauma‐informed care, empathic relating to consumers, training/education of staff, and team collaboration and cohesion are essential to reduction efforts. Attitudes towards elimination of containment methods were mixed, and so underlying all of these interventions should be a focus on challenging attitudes to containment as a means to prevent increases in injury rates (Goulet *et al*. 2017), and increasing staff reflection and communication regarding their individual attitudes towards seclusion/restraint and prevailing norms on their units.
